# Unmet need for family planning and associated factors among married women attending anti-retroviral treatment clinics in Dire Dawa City, Eastern Ethiopia

**DOI:** 10.1371/journal.pone.0250297

**Published:** 2021-04-16

**Authors:** Hiwot Dejene, Muluemebet Abera, Afework Tadele

**Affiliations:** 1 Department of Public Health, Mettu University, Mettu, Ethiopia; 2 Department of Population and Family Health, Jimma University, Jimma, Ethiopia; University of Mississippi Medical Center, UNITED STATES

## Abstract

**Background:**

Unmet need for family planning is a measure of the gap between women’s contraceptive behavior and their fertility desires. It should be measured among different population groups to effectively implement public health interventions. Thus, this study aimed to determine the magnitude of unmet need for family planning and associated factors among HIV- positive women in Dire Dawa city Anti-retroviral treatment (ART) clinics, Eastern Ethiopia.

**Methods:**

We carried out a facility-based cross-sectional study (March-June 2020) among 409 married women aged 15–49 on ART, using systematic random sampling. A face-to-face interview was done using a structured questionnaire. Bivariable and multivariable logistic regression was done to identify factors associated with unmet need for family planning.

**Results:**

Overall, 33% [95% confidence interval (CI): 28.9–37.9] of the respondents had unmet need for family planning. Woman’s residing in a rural area (adjusted odds ratio (AOR): 2.41 [95% CI: 1.24–4.67]), woman’s not attained formal education (AOR: 4.14 [95% CI: 1.73–9.93]) and attaining primary education (AOR: 2.97 [95% CI: 1.54–5.74]), poor knowledge (AOR: 2.87 [95% CI: 1.52–5.40]), and unfavorable attitude towards family planning (AOR: 2.21 [95% CI: 1.12–4.34]), clients not satisfied with family planning service (AOR: 6.34 [95% CI: 3.31–12.15]), the woman not having decision making power on family planning (AOR:3.97 [95% CI: 2.14–7.38]) and not getting family planning counseling in ART clinics (AOR: 2.87 [95% CI: 1.54–5.35]) were positively associated with having unmet need for family planning.

**Conclusion:**

This study indicates there a high unmet need for family planning among married HIV-positive women. Factors like a place of residence, educational status of women, knowledge and attitude towards family planning, client satisfaction with FP service, women decision-making power, and FP counseling service in ART. Therefore, we recommend that the concerned bodies should collaborate with ART clinics to design interventions that enhance access to family planning programs to combat the high unmet need for family planning among HIV-positive women.

## Introduction

The World Health Organization (WHO) defines unmet need for family planning (FP) as those women who are fecund and sexually active but do not use any method of contraception, and report not wanting any more children or wanting to delay the next child [1]. It measures the gap between women’s reproductive intentions and their contraceptive behavior. This indicator is useful for tracking progress towards the target of achieving universal access to reproductive health (RH) [2] by providing a measurement of the women’s ability in achieving their desired family size and birth spacing [3].

Unmet need for FP resulted in high fertility rates and eventually led to rapid population growth. In particular, for maternal and child health, rapid population growth has been seen as having a major public health impact [3]. In addition to rapid population growth, unintended pregnancy will occur as a result of unmet needs. It predisposes women to a variety of complications, including unsafe abortion, maternal death, malnutrition, and mental illness [4].

Pregnancy among HIV-positive women is a problem of public health concern owing to poor reproductive outcomes and new pediatric HIV infections causing much suffering and lifelong treatment as a result of scarce resources [5]. For HIV-positive women who do not want to become pregnant, FP is a proven, cost-effective method to prevent mother-to-child transmission of HIV (PMTCT) and, in turn, it reduces the number of children needing HIV treatment, care, and support [4, 6]. Also, effective FP use can bring new vertical HIV infections to almost zero by preventing unintended pregnancy [5]. That is why the WHO suggests reducing the unmet need for FP as a four-pronged approach for PMTCT as a cost-effective strategy [7].

Studies done among HIV positive women in different countries documented that there are high unmet needs for FP. For instance, in India (28%) [8], Gabon (28.7%), Ghana (27.8%), Uganda (45.1%), Nigeria (49%), and South Africa (28%) [9–13]. In Ethiopia, also different attempts were done to quantify the magnitude of unmet need among HIV-positive women; such as in Addis Ababa (25.1%), Hawassa (19.1%), and South Gondar (24.6%) [14–16].

Evidence from different studies presented that several factors hinder women from meeting their FP needs, especially in developing countries. These include age of woman, place of residence, educational status of the woman and her husband, existence of integration of ART and FP service, family size, duration of the marriage, HIV status of partner, and duration on ART [10, 17, 18].

Evaluating the magnitude of unmet need for FP and related factors will help to create successful RH programs for HIV-positive women. Particularly, among married women who are indicated to be less motivated than unmarried women to use contraception to prevent pregnancy and its complications [2]. The services may be used in the future to reduce accidental conception and mother-to-child transmission of HIV/AIDS. It is also used to enhance the existing limited body of knowledge regarding the topic. Even though various attempts have been made in Ethiopia to measure unmet need, only the magnitude but not the associated factors linked to the unmet need for FP was reported [16], and patients were only enrolled from a single residential setting (urban dwellers), tertiary and referral level facilities [14, 15] that lack evidence generalizability in the country’s circumstance.

To address this gap, investigating the various levels of facilities, in particular, primary health care facilities, and including rural residents is essential to keep up to date with current knowledge since the unmet need for FP is unique to the context and is influenced by cultural change [19]. In addition to the limited studies conducted in the country concerning the unmet need for FP among HIV-positive women, they were unable to document the link between the unmet need for FP and factors such as client satisfaction with service, women decision-making power at FP, attitudes toward FP, and the household wealth index among HIV-positive women [14, 15]. Even so, there is no study done in this study area previously. Therefore, this research was intended to determine the magnitude of unmet need and related factors by integrating all of the variables listed above that were not measured in the previous studies.

## Methods

### Study design and setting

A facility-based cross-sectional study was carried out from March 24 to June 12, 2020, at all public health facilities that provide ART service in Dire Dawa administrative city. Dire Dawa is located at a distance of 515 Kilometers from Addis Ababa, the capital city of Ethiopia in east direction. The Dire Dawa administrative council consists of Dire Dawa city and the surrounding rural areas. The public health facilities contain one referral hospital (Dilchora referral hospital), one primary hospital (Sabian primary hospital), and seven health centers (Number-one, Addis-ketema, Goro, Gende-kore, Legehare, Gende-gerada, and Dechatu).

### Participants

All HIV-positive married reproductive age (15–49) women attending ART clinics at a public facility in Dire Dawa city were the source population. All HIV-positive married reproductive age (15–49) women attending ART clinics at a public facility in Dire Dawa city during the study period and who had at least one previous visit at antiretroviral therapy clinics were eligible for the study.

### Sample size determination and sampling techniques

The sample size was calculated using the formula for the estimation of a single population proportion (n = [(Zα/2)^2^*P(1-P)]/d^2^) with the assumptions of 95% confidence level, marginal error (d) of 0.04, and 0.251 prevalence (P) of unmet need was taken from a previously conducted study [14]. Thus, after applying the finite population correction formula and adding 10% of the non-response rate, the final sample size obtained was 417. Then, the sample size was distributed using proportional allocation to size (PAS) to each antiretroviral therapy clinic in the city.

Finally, a systematic random sampling technique [K = N/n, yielding a sampling interval of five] was applied to select the study participants. In the interval, the first patient to be interviewed was chosen using the lottery method. Finally, the determined sample in each clinic was achieved through exit interviews with every K^th^ interval.

### Data collection procedures

Data were collected using a pretested structured questionnaire via face-to-face interviews. The data collection tool adapted from the revised unmet need for FP: Demographic Health Survey (DHS) analytical study 2012 [20] was used to estimate the magnitude of the unmet need for family planning among married HIV-positive women. Moreover, the questionnaire included questions related to factors associated with the unmet need for FP. Which are socio-demographic characteristics of the respondents, the socioeconomic status of the respondents, reproductive history, knowledge of FP, attitude toward FP, client satisfaction with the service, women’s decision-making power on FP, and health service factor. A medical record review was done to get HIV-related factors such as CD4 count, WHO stage, and duration on ART. Data were collected by trained nine Bachelor’s degree holder nurses that are worked in the ART clinics and supervised by two public health officers. Data collectors were assigned to data collection at each ART clinic and interview after the patient has taken routine care of the service.

### Measurements

**Fig 1:**
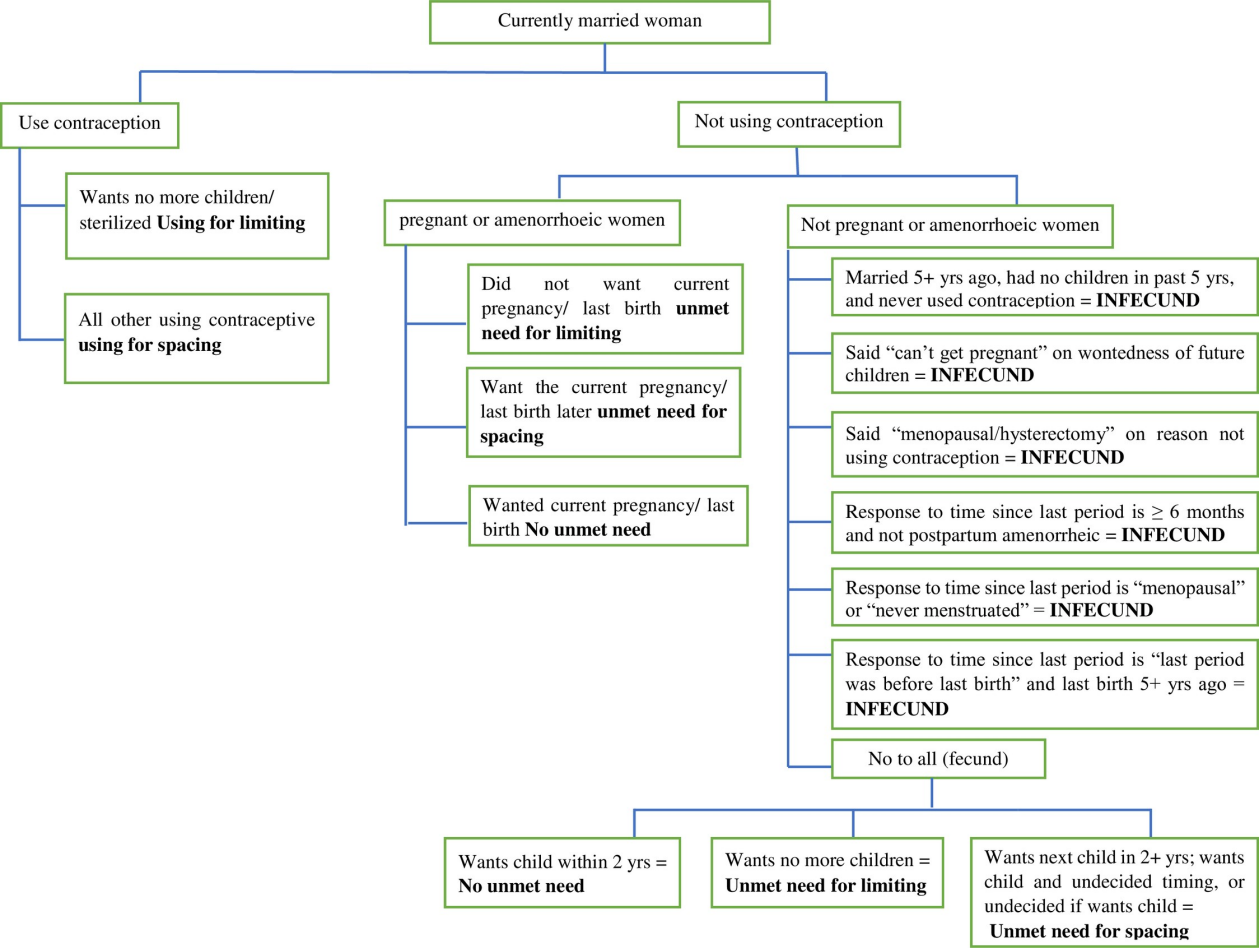
Revised unmet need algorithm.

Unmet need for FP among married HIV positive women was measured in this study by Revised definition of unmet need for currently married women [20]. The surveyed women were first divided into two groups, i.e., those who used contraceptives and those who didn’t use those methods. The nonusers were then subdivided into pregnant or amenorrhoeic women and non-pregnant or non-amenorrhoeic categorized at the time of the survey. The pregnant or amenorrhoeic women were further subdivided into three categories, i.e., those whose pregnancy was intended, mistimed, and unwanted at the time of the survey. Those who had mistimed and unwanted pregnancy was regarded as one component of the total unmet need. The other component consists of those who are neither pregnant nor amenorrhoeic. Further, they were divided into fecund or infecund; fecund women were again subdivided by their reproductive preference into 3 categories, i.e., those who want pregnancy soon (not included in unmet need), want no more pregnancy, and want pregnancy later (Fig 1). Knowledge about family planning was measured by using nine knowledge questions to construct a composite score. The first six questions have multiple responses and add each response from no answer to answering all options. The rest of the three questions are based on Yes and No by giving 1 to Yes and 0 to No. Based on the summation score, a score above 70% was considered as having good knowledge about family planning [21]. Attitude towards FP was measured by an attitude assessing tool using eight Likert scaled question items. Each item of the question has 5-points ranging from 1 (very unsatisfied) to 5 (very satisfied). A total score was calculated for each domain and transferred into ‘percent score’ by dividing the score with the possible maximum score and multiplying by 100. those who scored less than 85% were categorized as unfavorable whereas more or equal to 85% as favorable [22]. Married women decision-making power on modern FP use, scores have been developed for three sets of women: current users, Ever users, and None- users. **Current users**: six questions were asked to make a mean score. After computing the total, the score above the mean is said to have better decision-making power. **Ever uses**: Those who used modern FP at least once in their life time but currently not using; six questions were asked and the mean score above mean is said to have better decision-making power. **Non-users**: if their main reason for non- use is opposition from others the value was assigned as 0 and 1 if otherwise. Finally, married women ‘s decision-making in FP use among study units was set as a binary outcome variable by merging the three groups of women (woman’s only, Jointly with their husband and husband only) those scored mean and above the mean have better decision making power on modern FP use and who scored below the mean have less decision making power on modern FP use [23]. Client satisfaction with FP service was measured using fourteen Likert scaled question items. Each item of the question had 5-points ranging from 1 (very unsatisfied) to 5 (very satisfied) and finally, the mean score was computed to say women satisfied with the service. To apply the measurement first the women must be either ever user or current user [24]. The wealth index was measured by a simplified and updated Ethiopian wealth index equity tool. The tool contains 15 simplified household assets questions available from www.equitytool.org. The tool has an 84.2% agreement and 0.755 kappa statistics with the full Ethiopian Demographic Health Survey (EDHS,2016) wealth index measurement tool. Accordingly, the wealth index of the household was classified into three groups: poorest, middle and richest [25].

### Operational definitions and definition of terms

#### Integration of family planning and HIV services

Referred to a report from participants who received a family planning service in a spot with the ART services [26].

#### Demand for family planning

Refers to women with unmet need plus the percentage of currently using contraception (representing “met need”) [20].

### Data quality control

The questionnaire was translated to the local language (Amharic language) by a language expert and back-translated to English to guarantee consistency. The one-day training was given to the data collectors on the objective of the study, method of data collection, and ethical issues. The supervisors were also trained on how to monitor the data collection procedures. A pretest was done on 10% of the sample size in Hiwot Fana referral hospital to check the clarity and consistency of the questionnaires before the actual data collection. A reliability test was done and Cronbach’s alfa > 0.7 was taken for actual data collection. During data collection; each completed questionnaire was checked for completeness, clarity, and consistency at the site of data collection by the supervisors to take the corrective measure.

### Data processing and analysis

Data were entered into Epi Data version 3.1 and exported into SPSS version 25 for analysis. Data exploration was carried out to assess the completeness and descriptive statistics were used to describe the study participants’ data based on its nature. After checking multicollinearity by using VIF then binary logistic regression analysis was done to each independent variable with outcome variable to select candidate variables with P<0.25. Then entered into multivariable analysis to identify factors associated with the outcome variable and to control for confounders. Model fitness was checked by Hosmer & Lemeshow goodness of test (X^2^ = 14.41 with p-value 0.072). Backward stepwise logistic regression was used to determine factors independently associated with Unmet need. The adjusted odds ratio with a 95% confidence interval was computed to measure the strength of the association. Finally, reports were presented by the text, figures, and tables.

### Ethical consideration

Ethical clearance was obtained from the Institution Review Board (IRB) of institute of health of Jimma University. Written permission was obtained from the Dire Dawa administrative city Health Bureau. Then, heads of hospitals and health centers were communicated through formal letters from the city health office in addition to personal communication by the investigator. Informed written consent was obtained from each study participant before the interview. The interview was held in a separate room to maintain privacy and Answers to any questions were made completely confidential.

## Results

### Socio-demographic characteristics

A total of 409 married HIV-positive women participated in this study, with a response rate of 98.1%. The mean (SD) age of the women was 31.9 (5.6) years. Regarding the educational status of the respondent more than three fourth of the respondent (86.8%) and their husband (85.8%) attained formal education. Concerning the household wealth index class of the respondent, 38.4% of them were found under the highest class followed by the lowest class 31.5% (Table 1).

**Table 1:** Socio-demographic characteristics of married reproductive age women attending ART clinics in Dire Dawa administrative city Eastern Ethiopia, March-June 2020.

Variables	Categories	Frequency	Percentage (%)
**Age of the respondent**	15–24	47	11.5
	25–34	223	54.5
	35–49	139	34
**Duration of marriage**	< 5 years	62	15.2
	≥ 5 years	347	84.8
**Residence**	Urban	285	69.7
	Rural	124	30.3
**Religion status of the respondent**	Muslim	162	39.6
	Orthodox	149	36.4
	Protestant	92	22.5
	Catholic	6	1.5
**Educational status of the respondent**	No formal education	54	13.2
	Primary level education	118	28.9
	Secondary and above	237	57.9
**Educational status of the husband**	No formal education	58	14.2
	Primary level education	164	40.1
	Secondary and above	187	45.7
**Occupational status of the respondent**	Housewife	149	36.4
	Merchant	86	21.0
	Daily laborer	51	12.5
	Government employee	76	18.6
	Private employee	41	10.0
	Students	6	1.4
**Occupational status of her husband**	Merchant	80	19.6
	Daily laborer	54	13.2
	Government employee	112	27.4
	Private employee	144	35.2
	Others*	19	4.6
**Household wealth index**	Lowest wealth index	129	31.5
	Middle wealth index	123	30.1
	Highest wealth index	157	38.4

### Client related factors

All study participants had a previous history of using FP services. Nearly three fourth of the clients (72.9%) was satisfied with the FP service, and more than half of (54.4%) the respondents had better power to decide on the use of FP. Regarding knowledge and attitude towards FP indicated that near half of (43.8%) the respondent had poor knowledge and 1 in 3 women (35.7%) had a favorable attitude (Table 2).

**Table 2:** Client related factors of married reproductive age women attending ART clinics in Dire Dawa administrative city Eastern Ethiopia, March-June 2020.

Variable	Categories	Frequency	Percentage (%)
**Knowledge about FP**	Poor	230	56.2
	Good	179	43.8
**Attitude towards FP**	Unfavorable attitude	263	64.3
	Favorable attitude	146	35.7
**Client satisfaction with FP service**	Satisfied	298	72.9
	Dissatisfied	111	27.1
**Woman decision making power on FP**	less power	186	45.5
	better power	223	54.5

### HIV related factors

The mean (SD) duration of ART after starts was 6.58 (3.61). 4.9% of the participants was one year. The mean (SD) CD4 count of the respondent was 342 (149.24) and 24.7% of them were found under severe immune suppression. Only 9.5% of the respondents did not disclose their HIV status from their partner (Table 3).

**Table 3:** HIV related factors of married reproductive age women attending ART clinics in Dire Dawa administrative city Eastern Ethiopia, March-June 2020.

Variables	Categories	Numbers	Percentage %
**Duration of ART started**	≤3 years	85	20.8
	> 3 years	323	79.2
**Presence of opportunistic infection (OI)**	Yes	126	30.8
	No	283	69.2
**Partners tested**	Yes	349	85.3
	No	28	6.8
	I don’t know	32	7.8
**Disclosure status**	Yes	370	90.5
	No	39	9.5
**Partner HIV status**	Positive	284	81.4
	Negative	65	18.6

### Health service-related factors

**Fig 2:**
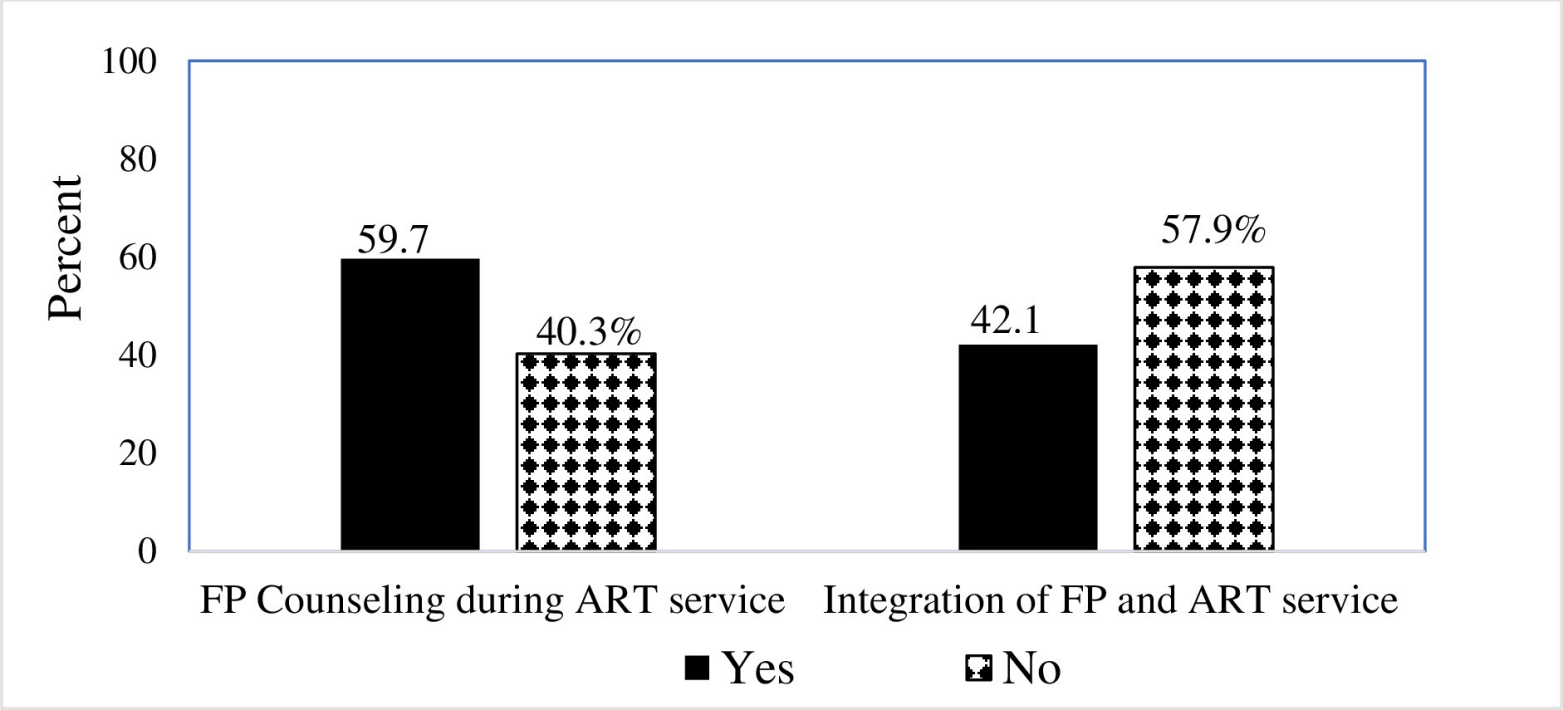
Health service-related factors among HIV-positive women.

More than half of (57.9%) the respondents have not received integration of service in the ART clinics. In contrast to this more than half of (59.7%) the respondent was received FP counseling service in the ART clinics (Fig 2).

### Unmet need for family planning

**Fig 3:**
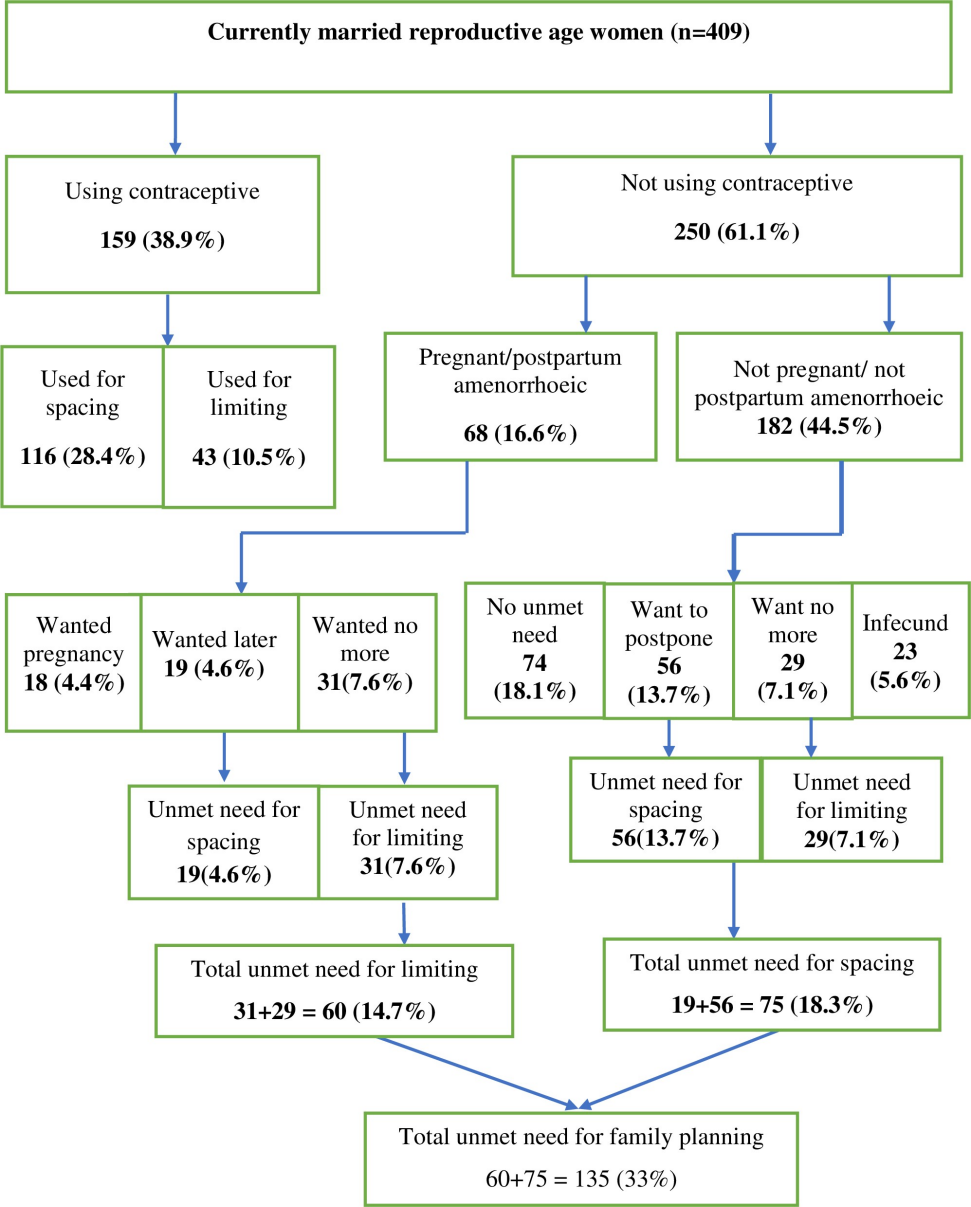
Schematic diagram of unmet need for FP among HIV-positive women.

The total unmet need for FP among married women attending ART clinics in Dire Dawa administrative city was found to be 135 (33%) [95% CI: 28.9–37.9] of which 75 (18.3%) for spacing and 60 (14.7%) for limiting. The total demand for FP among married women attending ART clinics in Dire Dawa city was 71.9% (current use 38.9% + unmet need 33%) (Fig 3).

### Perceived reasons for not using family planning methods

**Fig 4:**
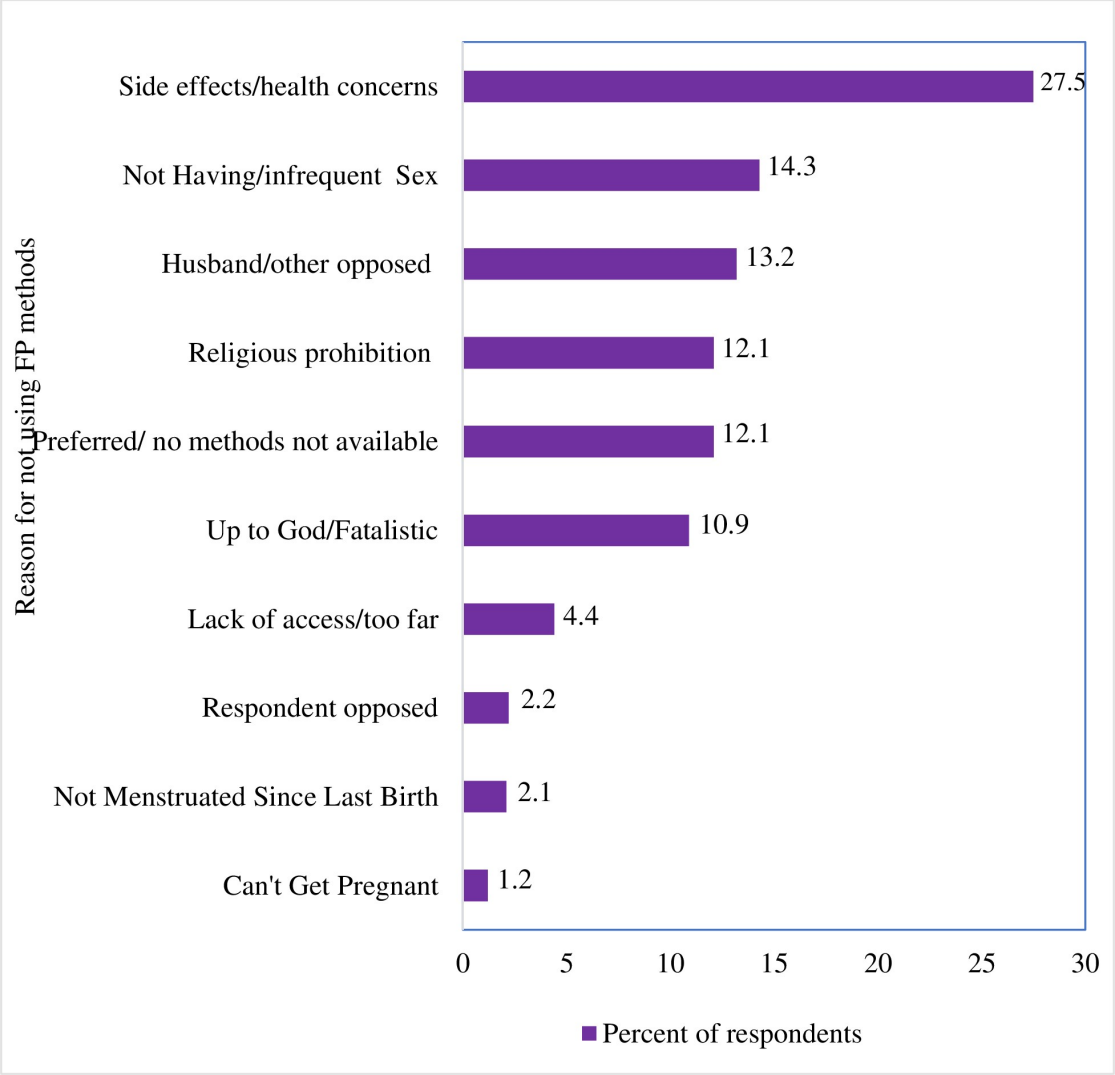
Perceived reasons for not using FP methods among HIV-positive women.

Out of the total 182 women not using FP, only 85 women (of which six of them respond to two responses at once) required FP but didn’t use the service. The reason stated for not FP was 40 (43.9%) of respondents due to method-related reasons followed by 26 (28.5%) fertility-related reasons and 25 (27.5%) opposition to using for different reasons (Fig 4).

### Factors associated with unmet need for family planning

In bivariable analysis indicated that sociodemographic variables (place of residence, educational status of the woman, and educational status of the husband), client-related factors (knowledge about family planning, attitude towards family planning, client satisfaction with family planning service, and woman decision making power on family planning), HIV related factors (duration on ART, partner tested and disclosure status) and health service-related factors (counseling service in ART service and integration of FP service with ART) was a candidate for multivariable logistic regression analysis. In multivariable logistic regression analysis, the variables: place residence [AOR: 2.41, 95% CI: 1.24–4.67], educational status of a woman(woman not attending formal education [AOR: 4.14, 95% CI: 1.73–9.93] and woman attending primary level education [AOR: 2.97, 95% CI: 1.54–5.74]), knowledge about FP [AOR: 2.87, 95% CI: 1.52–5.40], attitude towards FP [AOR: 2.21, 95% CI: 1.12–4.34], client satisfaction with the FP service [AOR: 6.34, 95% CI: 3.31–12.15], woman decision making power [AOR:3.97, 95% CI: 2.14–7.38], and FP counseling service in ART clinics [AOR: 2.87, 95% CI: 1.54–5.35] were independently associated with unmet need for FP (S2 File).

## Discussion

This study assessed the magnitude and factors related to unmet need for FP among married women attending ART clinics in Dire Dawa administrative city. According to the study findings, the unmet need for FP was 33% of which was 18.3% for spacing and 14.7% for limiting. Place of residence, educational status of the respondent, knowledge about FP, attitude toward FP, client satisfaction with FP service, women decision making power, and FP counseling service in ART clinics were the independent predictors of unmet need for FP among married women attending ART clinics.

This research found that unmet need for FP (33%) to be consistent with the previous study in Addis Ababa, where the unmet need for FP among HIV-positive women was 31.1% [27], and in India, where 28.% of ART attending women had an unmet need for FP [8]. However, the result in this study is higher than the study in Ethiopia, as was the study in Hawassa (19.1%) [15] and Addis Ababa (25.1%) [14]. The potential cause of the variance of the outcome may be due to dissimilarity in the sample population of married and union women, the other possibility is that the present study may have rural residents as participants in the study. As well as the present report, all ART clinics located in the city are included in contrast to prior surveys of referral hospital attendants.

In comparison to the above, this research was lower than the Uganda study found that the prevalence of unmet need in the clinic was 45.1% [11]. This might be due to the lack of FP onsite service in the facility. This reason might increase the risk of having unmet need for FP [11]. There is also a strategy in Ethiopia that has been used to reduce the magnitude of unmet needs by recognizing women who have not met the need for FP by developmental women’s armies and connecting them to health facilities for information, education, counseling, and services [28].

Women live in a rural area was significantly associated with unmet need for FP. This result in line with other studies in Tigray [29], Nigeria [30], and India [8]. The possible explanation might be even if FP knowledge was even distributed in all residential areas, women living in rural areas were less exposed to messaging regarding FP [31].

HIV-positive women who did not attend formal education and only did attend primary education were heightened the risk of having unmet need for FP as compared to those who had attended secondary education and above. This finding is consistent with a study done in Hawassa (21) and Nigeria [30]. This might be due to education is the prime influencing factor and it affects the attitudinal and behavioral patterns of the individuals [32].

Poor knowledge about FP has increased the risk of having unmet need for FP. The finding is supported by a study done in Nigeria [30]. Unfavorable attitude toward FP also increased the risk of having unmet need for FP. It is consistent with research conducted among women of reproductive age in southern nations nationalities and peoples’ area, Ethiopia [33], and Juba District, South Sudan [34]. The possible explanation might be attitude is a notional concept and although it cannot be observed directly, the effects on behavior are well known as used as key factors to start behaving and maintaining it continuously. Due to this reason, a woman who had a favorable attitude towards FP was more utilize in the service than a woman who had an unfavorable attitude.

Client satisfaction has been described as a key to the decisions of the clients to use and continue to use services or willingness to return for future services. This research also showed that a client dissatisfied with FP services was heightened the risk of having unmet need for FP. This finding supported by DHS data shows that between 7 and 27% of women stop using a contraceptive method for reasons related to the service environment, including service quality, availability of a sufficient choice of methods, commodity stock-outs, and ineffective referral mechanisms [35].

A woman who had less power to decide about the use of FP was at high risk of having an unmet need for FP. This finding was comparable with a study done in Addis Ababa [14]. In women’s decision-making power on FP behind there is an involvement of male partners in the decision of FP utilization. This might result in the active involvement of male and female in FP decisions to increase the utilization rate of FP and decrease unmet need as compared to decision making power only made by male/husband.

Family planning counseling in ART clinics was significantly associated with unmet need for FP. Counseling is important to help women and their partners to gain increased control over their reproductive health and it also increases FP uptake, continuation and is essential for ensuring informed and voluntary decision making. The finding in Addis Ababa [14] also support this.

### Limitation of the study

The cross-sectional nature of the study design does not allow causality ascertainment. Moreover, since these pregnant and postpartum amenorrhoeic women were questioned about their present and previous pregnancy, social desirability bias and memory bias may exist. Since they do not recall or purposely mask specifically whether or not the pregnancy was desired. This study was also limited to married women only and would therefore not be generalized to unmarried women with unmet FP needs in HIV-positive women.

## Conclusion

This study indicates there is a high unmet need for FP among married HIV-positive women according to the national reproductive health strategy target [36]. Difficulties that increase women risk to have unmet need for family planning were women attain low educational status, have poor knowledge and unfavorable attitude towards family planning, dissatisfied with FP service and do not get counseling about family planning in ART clinics, and also lacking decision making power to use family planning. Therefore, we recommend the concerned bodies should collaborate with ART clinics to design interventions that enhance access to family planning program to combat the high unmet need for family planning among HIV positive women.

## Supporting information

S1 File (DOCX)

S2 File (DOC)

S1 Dataset (XLS)

## Abbreviations

AOR: Adjusted Odds Ratio
ART: anti-retroviral treatment
CI: Confidence Interval
COR: Crude Odds Ratio
FP: family planning
