# Supplementary material for: Unmet need for family planning and associated factors among married women attending anti-retroviral treatment clinics in Dire Dawa City, Eastern Ethiopia
Source: PLoS One. 2021 Apr 16;16(4):e0250297. doi: 10.1371/journal.pone.0250297 (PMC8051792; doi:10.1371/journal.pone.0250297)
Supplement: S1 File — (DOCX) [file pone.0250297.s001.docx]

# English version questionnaires

| **Code** | ______________________ | | |
| --- | --- | --- | --- |
| s.no | Question | Code and categories | Skip |
| **Part I socio-demographic characteristics of the respondent** | | | |
| 101 | Age in completed years | ________________ |  |
| 102 | Duration of marriage | _________________ |  |
| 103 | Place of residence | 1. Urban 2. Rural |  |
| 104 | Religion | 1. Muslim 2. Orthodox 3. Protestant 4. Catholic 5. Other _________ |  |
| 105 | Educational status of the respondent | 1. No education 2. Read & write, but no formal education 3. Grade _________ |  |
| 106 | Partner’s educational status | 1. No education 2. Read & write, but no formal education 3. Grade ________ |  |
| 107 | Occupational status of the respondent | 1. House wife 2. Merchant 3. Daily laborer 4. Government employee 5. Private employee 6. student 7. other _________ |  |
| 108 | Occupational status of her partner | 1. Merchant 2. Daily laborer 3. Government employee 4. Private employee 5. student 6. others(specify) |  |
| **Part II Reproductive history of the respondent** | | | |
| 201 | Age of the first marriage | ___________ |  |
| 202 | Have you ever been pregnant? | 1. Yes 2. No | If no Go to 208 |
| 203 | How many pregnancies have you had? | ________ |  |
| 204 | How many live children do you have? | ________ |  |
| 205 | Do you have experience of child loss? | 1. Yes 2. No |  |
| 206 | Have you ever experienced a pregnancy terminated with abortion? | 1. Yes 2. No | If no go to 208 |
| 207 | If the answer to Q 206 is yes, how many times? | __________ |  |
| 208 | How many children would you like to have in your life? | __________ |  |

| **Part III: Questions and Filters for Unmet Need Definition** | | | |
| --- | --- | --- | --- |
| **s.no** | **Questions and filters** | **Coding categories** | **Skip** |
| 301 | Are you pregnant now? | 1. Yes |  |
|  |  | 1. No | Go to 304 |
|  |  | 1. Unsure |  |
| 302 | When you got pregnant, did you want to get pregnant at that time? (current) | 1. Yes | Go to 304 |
|  |  | 1. No |  |
| 303 | Did you want to have a baby later on or did you not want any (more)  children? | 1. Later |  |
|  |  | 1. No more |  |
| 304 | When did your last menstrual period start?  ________________________  (date if given) | 1. Days ago, _______ 2. Weeks ago, _____ 3. Months ago, _____ 4. Years ago, ______ 5. In menopause or had hysterectomy 6. Before last birth 7. Never menstruated |  |
| 305 | Check 301  Not pregnant or unsure pregnant | | Go to 307 |
| 306 | Are you currently doing something or using any method to delay or avoid getting pregnant? | 1. Yes 2. No |  |
| 307 | Have you ever used anything or tried in any way to delay or avoid getting pregnant? | 1. Yes 2. No |  |
| 308 | What name was given to your (last) baby? RECORD NAME | Name _____________ |  |
| 309 | In what month and year was (NAME) born?  PROBE: When is his/her birthday? | Month ________  Year __________ |  |
| 310 | Check 309  Birth within 5 years or later Birth before 5 years | | Go to 314 |
| 311 | When you got pregnant with (NAME), did you want to get pregnant at that time? | 1. Yes | Go to 313 |
|  |  | 1. No |  |
| 312 | Did you want to have a baby later on, or did you not want any (more)  children? | 1. Later 2. No more |  |
| 313 | Has your menstrual period returned since the birth of (NAME)? | 1. Yes 2. No |  |
| 314 | Now I would like to ask about your (first) (husband/partner). In what month and year did you start living with him? | Months _________  Don’t know months  Years __________  Don’t know months |  |
| 315 | When was the last time you had sexual intercourse?  If less than 12 months, answer must be recorded in days, weeks or months.  If 12 months (one year) or more, answer must be Recorded in years. | 1. Days ago, ______ 2. Weeks ago, _____ 3. Months ago, _____ 4. Years ago, _______ |  |
| 316 | Now I have some questions about the future. After the child you are expecting now, would you like to have another child, or would you prefer not to have any more children? | 1. Have another child | Go to 319  Go to 401 |
|  |  | 1. No more |  |
|  |  | 1. Undecided/ I don’t know |  |
| 317 | Now I have some questions about the future. Would you like to have another child, or would you prefer not to have any (more) children? | 1. Have another child |  |
|  |  | 1. No more child | Go to 321 |
|  |  | 1. Says she can’t get pregnant | Go to 401 |
|  |  | 1. Undecided/ don’t know |  |
| 318 | Check 301  **Not pregnant or unsure**  (How long would you like to wait from now before the birth of another child?)  **Pregnant**  (After the birth of the child you are  expecting now, how long would  you like to wait before the birth of  another child?) | 1. Months 2. Years |  |
|  |  | 1. Soon now 2. Says she can’t get pregnant 3. After marriage 4. Other 5. Don’t know | Go to401 |
| 319 | Check 301  Not pregnant or unsure pregnant | | Go to 401 |
| 320 | Check 306  Not currently using methods using methods | | Go to 401 |
| 321 | Check 318  Not Asked 2years or more years with in 2 years or less | | Go to 401 |
| 322 | Check 317  Want to have another child Want no more or none | |  |
|  | You have said that you do not want (a/another) child soon.  Can you tell me why you are not using a method to prevent pregnancy?  Any other reason? | You have said that you do not want any (more) children.  Can you tell me why you are not using a method to prevent pregnancy?  Any other reason? |  |
|  | Recode all mentioned reasons  **Fertility-Related Reasons**   1. Not Having Sex 2. Infrequent Sex 3. Menopausal/Hysterectomy 4. Can't Get Pregnant 5. Not Menstruated Since Last Birth 6. Breastfeeding 7. Up to God/Fatalistic   **Method-Related Reasons** **Opposition to use**  8. Side Effects/ Health Concerns 18. Respondent Opposed  9. Lack of Access/Too Far 19. Husband/Partner Opposed 10. Costs Too Much 20. Others Opposed  11.Preferred Method not available 21. Religious Prohibition  12. No Method Available  13.Inconvenient to Use  14.Interferes with Body's  15.Normal Processes  16.Other _______________________  17.Don't know | |  |

| **Part IV: Knowledge about contraceptives** | | | |
| --- | --- | --- | --- |
| 401 | Have you ever heard of family planning? | 1. Yes 2. No | If no Go to 501 |
| 402 | What is your source of information about family planning? thick all mentioned | 1. Mass media (Radio, Television)  2. Husband  3. Peer  4. Health professionals  5. Other specify________ |  |
| 403 | Which type of modern FEMALE  contraceptive methods do you  know? /multiple response is  allowed/ | 1. Female sterilization 2. Pills 3. Injectable 4. Implants 5. Emergency contraception 6. Female condom 7. IUD   Other (specify)_______ |  |
| 404 | Do you know if there is any family planning method for men? | 1. Yes 2. No | If no Go to 406 |
| 405 | Which type of MALE contraceptive methods do you know? (multiple responses possible) | 1. Condom 2. Vasectomy/male sterilization 3. Others specify _____ |  |
| 406 | Which advantages of FP do you know? /multiple responses allowed mark all that apply/ | 1. To Limit family size 2. To avoid unwanted pregnancy 3. To Space childbirth 4. For the mothers/child health 5. I don’t know   6. Other specify _______ |  |
| 407 | Between two consecutive children, how many years of intervals do you think is good? (How long they should be spaced) | 1. Less than one year 2. One to two years 3. Three to five years 4. I don’t know 5. Other specify _____ |  |
| 408 | Do you know where to get modern contraceptive methods? | 1. Yes 2. No | If No go to 501 |
| 409 | Which one do you know? /mark all that you know/ | 1. Health center 2. Health post 3. Pharmacy 4. Hospital 5. Social markets 6. Private clinics 7. At home from CBD and outreach service agents 8. Others, specify_______ |  |

| **Part V: attitude towards family planning** | | | | | | | |
| --- | --- | --- | --- | --- | --- | --- | --- |
| **s.no** | **Questions** | | **Very disagree** | **Disagree** | **Neutral** | **Agree** | **Very agree** |
| 501 | | Pregnancy must be properly planned and not just allow it to happen on its own |  |  |  |  |  |
| 502 | A mother who has just delivered and her husband should be given adequate information regarding family planning | |  |  |  |  |  |
| 503 | Pregnancy should be planned and discussed together between husband and wife | |  |  |  |  |  |
| 504 | Pregnancy which is too closely spaced should be avoided by using family planning method | |  |  |  |  |  |
| 505 | Husband should involve during the planning and ensuring the use of contraceptive method by his wife | |  |  |  |  |  |
| 506 | Modern contraceptive method is more effective than traditional method | |  |  |  |  |  |
| 507 | The use of contraceptive method will not interfere sexual relationship between husband and wife | |  |  |  |  |  |
| 508 | Support from husband is important to determine the success of family planning program | |  |  |  |  |  |
| **Part VI: client satisfaction with family planning service** | | | | | | | |
| **Remember**: to answer the following section the woman must be either ever user or current user | | | | | | | |
| **s.no** | **Questions** | | **Very dissatisfied** | **Dissatisfied** | **Neutral** | **Satisfied** | **Very satisfied** |
| 601 | Registration staff warmly welcomed you | |  |  |  |  |  |
| 602 | Professionals informed you where FP service department | |  |  |  |  |  |
| 603 | Professionals were available when required | |  |  |  |  |  |
| 604 | Professionals introduce their name to you | |  |  |  |  |  |
| 605 | Professionals spent enough time in consultation | |  |  |  |  |  |
| 606 | Professionals were respectful | |  |  |  |  |  |
| 607 | Professionals performs the procedure with cleanliness and sanitation | |  |  |  |  |  |
| 608 | Professionals explanation was clear and straightforward | |  |  |  |  |  |
| 609 | Choice of methods available | |  |  |  |  |  |
| 610 | Professionals gave adequate information | |  |  |  |  |  |
| 611 | Health facility easily accessible | |  |  |  |  |  |
| 612 | Location of the family planning service department | |  |  |  |  |  |
| 613 | The waiting room has enough sitting chairs | |  |  |  |  |  |
| 614 | Cleanliness of the health facility | |  |  |  |  |  |

| **Part VII: women decision making power on family planning** | | | | | |
| --- | --- | --- | --- | --- | --- |
| Now I will ask about your decision-making power on family planning | | | | | |
| **S,no** | **Questions** | | **Joint decision** | **Husband only** | **Wife only** |
| 701 | | Who decide on the use of family planning? |  |  |  |
| 702 | | Who decide on number of children |  |  |  |
| 703 | | Who decide on Choice of family planning methods |  |  |  |
| 704 | | Who decide on place of family planning service provision |  |  |  |
| 705 | | Who decide on when to give birth |  |  |  |
| 706 | | Who decided on where to give birth |  |  |  |

| **Part VIII:** **Information on HIV related variables** | | | |
| --- | --- | --- | --- |
| **s.no** | **Questions** | **Response** | **Skip** |
| 801 | How long has it been since you started ART treatment? | _____________ |  |
| 802 | What is your current CD4 count? | _____________ cells/mm^2^ (from card) |  |
| 803 | What is your current WHO stage | ____________ (from card) |  |
| 804 | Is there presence of opportunistic infection | 1. Yes 2. No (from card) |  |
| 805 | Has your partner got HIV tested? | 1. Yes | Go to 806 |
|  |  | 1. No | Go to 807 |
|  |  | 1. I don’t know | Go to 807 |
| 806 | If Yes to Q 806 what is the HIV status of your partner? | 1. Positive 2. Negative 3. No response 4. I don’t know |  |
| 807 | Your husband knows your HIV status | 1. Yes 2. No |  |
| 808 | Do you get family planning counseling during ART service provision | 1. Yes 2. No |  |
| 809 | Do you get Family planning services in ART clinic | 1. Yes 2. No |  |
| **Part IX: survey questions in full wealth index** | | | |
| 901 | Does your household have electricity? | 1. Yes 2. No |  |
| 902 | Does your household have a radio? | 1. Yes 2. No |  |
| 903 | Does your household have a television? | 1. Yes 2. No |  |
| 904 | Does your household have a refrigerator? | 1. Yes 2. No |  |
| 905 | Does your household have an electric mitad? | 1. Yes 2. No |  |
| 906 | Does your household have a table? | 1. Yes 2. No |  |
| 907 | Does your household have a chair? | 1. Yes 2. No |  |
| 908 | Does your household have a bed with a cotton/ sponge/ spring mattress? | 1. Yes 2. No |  |
| 909 | Does any member of this household have a bank account? | 1. Yes 2. No |  |
| 910 | What is the main source of drinking water for members of your household? | 1. Piped to yard/plot 2. Other |  |
| 911 | What kind of toilet facility do members of your household usually use? | 1. Pit latrine without slab / open pit 2. No facility / bush / field 3. Other |  |
| 912 | What type of fuel does your household mainly use for cooking? | 1. Electricity 2. Wood 3. Other |  |
| 913 | What is the main material of the floor in your household? | 1. Earth/sand 2. Other |  |
| 914 | What is the main material of the exterior walls in your household? | 1. Bamboo with mud 2. Other |  |
| 915 | What is the main material of the roof in your household? | 1. Metal / corrugated iron 2. Other |  |
| **Thank you !!!** | | | |
