# Supplementary material for: Unmet need for family planning and associated factors among married women attending anti-retroviral treatment clinics in Dire Dawa City, Eastern Ethiopia
Source: PLoS One. 2021 Apr 16;16(4):e0250297. doi: 10.1371/journal.pone.0250297 (PMC8051792; doi:10.1371/journal.pone.0250297)
Supplement: S2 File — (DOC) [file pone.0250297.s002.doc]

Independent predictors of unmet need for family planning among married reproductive age women attending ART clinics in Dire Dawa administrative city Eastern Ethiopia,2020.

| **Variable** | **Categories** | **Unmet need for FP** | | **COR (95% CI)** | **P-value** | **AOR (95% CI)** | **P-value** |
| --- | --- | --- | --- | --- | --- | --- | --- |
|  |  | Yes (%) | No (%) |  |  |  |  |
| **Residence** | Urban | 70 (24.6) | 215 (75.4) | 1 |  | 1 |  |
|  | Rural | 65 (52.4) | 59 (47.6) | **3.38 [2.17-5.27] **** | **0.000** | **2.41 [1.24-4.67] *** | **0.004** |
| **Educational status of the respondent** | No formal education | 29 (53.7) | 25 (46.3) | **5.55 [2.95-10.43] **** | **0.000** | **3.23 [1.28-8.07] *** | **0.014** |
|  | Primary education | 65 (55.1) | 53 (44.9) | **5.86 [3.57-9.62] **** | **0.000** | **2.32 [1.18-4.58] *** | **0.009** |
|  | Secondary and above | 41 (17.3) | 196 (82.7) | 1 |  | 1 |  |
| Educational status of the husband | No formal education | 30 (51.7) | 28 (48.3) | 4.49 [2.39-8.44] ** | 0.000 | 0.98[0.28-3.32] | 0.932 |
|  | Primary education | 69 (42.1) | 95 (57.9) | 3.05 [1.89-4.91] ** | 0.000 | 0.59 [0.28-1.30] | 0.597 |
|  | 2^nd^ & above education | 36 (19.3) | 151 (80.7) | 1 |  | 1 |  |
| **Knowledge about FP** | Poor | 112 (48.7) | 118 (51.3) | **6.44 [3.87-10.70] **** | **0.000** | **2.82 [1.48-5.37] *** | **0.000** |
|  | Good | 23 (12.8) | 156 (87.2) | 1 |  | 1 |  |
| **Attitude towards FP** | Unfavorable attitude | 111 (42.2) | 152 (57.8) | **3.71 [2.24-6.12] **** | **0.000** | **2.21 [1.12-4.34] *** | **0.008** |
|  | Favorable attitude | 24 (16.4) | 122 (83.6) | 1 |  | 1 |  |
| **Client satisfaction** | Satisfied | 61 (20.5) | 237 (79.5) | 1 |  | 1 |  |
|  | Dissatisfied | 74 (66.7) | 37 (33.3) | **7.77 [4.79-12.62] **** | **0.000** | **6.34 [3.31-12.15] **** | **0.000** |
| **Women decision making power** | Has less power | 101 (54.3) | 85 (45.7) | **6.61 [4.15-10.52] **** | **0.000** | **3.97 [2.14-7.38] **** | **0.000** |
|  | Has better power | 34 (15.2) | 189 (84.8) | 1 |  | 1 |  |
| Duration on ART | ≤3 years | 23 (27.1) | 62 (72.9) | 1 |  | 1 |  |
|  | > 3 years | 112 (34.7) | 212 (65.3) | 1.42 [0.84-2.43] | 0.186 | 1.43 [0.59-3.43] | 0.163 |
| Partner tested | Yes | 107 (30.7) | 242 (69.3) | 1 |  | 1 |  |
|  | No | 13 (46.4) | 15 (53.6) | 1.96 [0.90-4.26] | 0.089 | 0.79 [0.17-3.72] | 0.761 |
|  | I don’t know | 15 (46.9) | 17 (53.1) | 1.99 [0.96-4.14] | 0.064 | 1.19 [0.1-4.55] | 1.480 |
| Disclosure status | Yes | 112 (30.3) | 258 (69.7) | 1 |  | 1 |  |
|  | No | 23 (59.0) | 16 (41.0) | 3.31 [1.69-6.51] ** | 0.001 | 1.35 [0.47-3.84] | 1.294 |
| **FP counseling in ART** | Yes | 54 (22.1) | 190 (77.9) | 1 |  | 1 |  |
|  | No | 81 (49.1) | 84 (50.9) | 3.39 [2.21-5.21] ** | **0.000** | **2.87 [1.54-5.35] *** | **0.003** |
| Integration of FP and ART service | Yes | 34 (19.8) | 138 (80.2) | 1 |  | 1 |  |
|  | No | 101 (42.6) | 136 (57.4) | 3.01 [1.91-4.75] ** | 0.000 | 1.76 [0.88-3.52] | 0.142 |

****** significant at p-value <0.001 ***** significant at p-value <0.05 1=references
